# A broad survey of hydraulic and mechanical safety in the xylem of conifers

**DOI:** 10.1093/jxb/eru218

**Published:** 2014-06-10

**Authors:** Pauline S. Bouche, Maximilien Larter, Jean-Christophe Domec, Régis Burlett, Peter Gasson, Steven Jansen, Sylvain Delzon

**Affiliations:** ^1^Institute for Systematic Botany and Ecology, Ulm University, Ulm, Germany; ^2^INRA, UMR BIOGECO, F-33610 Cestas, France; ^3^University of Bordeaux, UMR BIOGECO, 33405 Talence, France; ^4^Bordeaux Sciences Agro, University of Bordeaux, 33175 Gradignan, France; ^5^INRA, UMR TCEM, F-33140 Villenave d’Ornon, France; ^6^Jodrell Laboratory, Royal Botanic Garden, Kew, Richmond, Surrey, TW9 3DS, UK

**Keywords:** Cavitation resistance, hydraulic efficiency, mechanical strength, seal capillary–seeding, torus–margo pit, xylem anatomy, wall implosion.

## Abstract

Drought-induced forest dieback has been widely reported over the last decades, and the evidence for a direct causal link between survival and hydraulic failure (xylem cavitation) is now well known. Because vulnerability to cavitation is intimately linked to the anatomy of the xylem, the main objective of this study was to better understand the xylem anatomical properties associated with cavitation resistance. An extensive data set of cavitation resistance traits and xylem anatomical properties was developed for 115 conifer species, with special attention given to the micro-morphology of bordered pits. The ratio of torus to pit aperture diameter, so-called torus overlap, increased with increasing cavitation resistance, while the flexibility of the margo does not seem to play a role, suggesting that air-seeding is located at the seal between the aspirated torus and pit aperture. Moreover, punctured tori were reported in various Pinaceae species. Species resistant to cavitation had thicker tracheid walls, while their lumen diameter (conduit size) was only slightly reduced, minimizing the impact on hydraulic conductance. The results also demonstrated (i) the existence of an indirect trade-off between hydraulic safety and mechanical strength; and (ii) a consistency between species distribution and xylem anatomy: species with a wide torus overlap and high valve effects are found in arid environments such as the Mediterranean region.

## Summary

Torus overlap and tracheid wall thickness are strongly correlated with cavitation resistance based on data from 115 conifer species.

## Introduction

Evidence for drought-induced forest dieback has been reported worldwide (Bigler *et al.*, 2007; Van Mantgem *et al.*, 2009; Allen *et al.*, 2010; Peng *et al.*, 2011; Sanchez-Salguero *et al.*, 2012). There is growing evidence that all forest types or climate zones are equally vulnerable to drought events, even in currently mesic environments (Allen *et al.*, 2010; Choat *et al.*, 2012). Although gymnosperms seem to be on average more resistant to cavitation (Maherali *et al.*, 2004; S. Delzon *et al*., unpublished data) and have greater hydraulic safety margins *per se* than angiosperms (Choat *et al.*, 2012), they are also not immune to drought-induced mortality (Breshears *et al.*, 2005; Sanchez-Salguero *et al.*, 2012; Hartmann *et al.*, 2013).

Resistance to cavitation is a crucial trait in trees to cope with drought stress (Cochard and Delzon, 2013) in addition to, for example, rooting depth, internal water storage, and changes in biomass allocation or leaf anatomy. Indeed, substantial evidence of a direct causal link between drought resistance and cavitation resistance has been highlighted in both conifers (Brodribb and Cochard, 2009; Brodribb *et al.*, 2010) and angiosperms (Barigah *et al.*, 2013; Urli *et al.*, 2013). Global surveys of cavitation resistance in woody species have not surprisingly shown that species from xeric climates are more resistant to embolism than species from wet climates (Maherali *et al.*, 2004; Cochard *et al.*, 2008; Choat *et al.*, 2012). Incorporation of phylogenetic information strengthened these adaptive inferences and suggests that cavitation resistance-related traits are under natural selection (Maherali *et al.*, 2004; Willson *et al.*, 2008; Pittermann *et al.*, 2010). Hence, there is convincing evidence that the geographical distribution of many tree species is determined by their ability to resist drought-induced embolism (Engelbrecht *et al.*, 2007; Choat *et al.*, 2012; Delzon and Cochard, 2014).

Drought-induced embolism occurs in the xylem, in which water is transported under tension (Tyree and Zimmerman, 2002). While the exact mechanisms remain unknown, drought-induced embolism formation is thought to occur via air leakage from an embolized conduit (non-functional) to a functional conduit. When the pressure in the xylem is sufficiently negative, the rupture of an air–sap meniscus allows propagation of air bubbles through porous interconduit pit membranes (Crombie *et al.*, 1985; Cochard *et al.*, 1992; Jarbeau *et al.*, 1995; Tyree and Zimmerman, 2002). Cavitation resistance would therefore be influenced by the structure and function of bordered pits. Accordingly, variation in xylem anatomy, conduit characteristics, and bordered pits has been associated with cavitation resistance in both angiosperms (Sperry and Hacke, 2004; Jansen *et al.*, 2009; Lens *et al.*, 2011) and conifers (Hacke *et al.*, 2004; Domec *et al.*, 2006; Delzon *et al.*, 2010).

Cavitation in angiosperms occurs by air-seeding at the pit membrane level. Under well-hydrated conditions, the pit membrane separating two functional vessels is in a relaxed position (i.e. unaspirated) and sap flows through the pores of the membrane. When water stress occurs, the pressure difference between an embolized and functional vessel leads to the rupture of an air–sap meniscus located within the pit. Embolism formation in angiosperms seems to depend on the size of the largest pores in the pit membranes (Choat *et al*., 2003, 2004; Christman *et al.*, 2009). In conifers, the intertracheid pits are morphologically characterized by a centrally located torus and a porous margo region. When the pressure difference in xylem increases, the deflection of the torus against the pit aperture seals off the embolized tracheid (Liese and Bauch, 1967; Bailey, 1913). This so-called ‘valve effect’ prevents the spread of air into the functional xylem and may depend on the torus diameter relative to pit aperture diameter (Hacke *et al.*, 2004; Domec *et al.*, 2006; Delzon *et al.*, 2010).

How does cavitation occur in torus–margo pits? Different mechanisms of air-seeding have been proposed to explain cavitation in conifers (for a review, see Cochard, 2006). Recent studies demonstrated that as for angiosperms, cavitation occurs by rupture of an air–sap meniscus in the vicinity of the pit membrane (Sperry and Hacke, 2004; Cochard *et al.*, 2009), but the exact location of where the meniscus breaks is unknown. Two mechanisms are likely: (i) air bubbles pass through pores at the edge of the torus when the torus and the inner wall of the pit membrane are not perfectly sealed (seal capillary-seeding hypothesis; Cochard *et al.*, 2009; Delzon *et al.*, 2010; Pittermann *et al.*, 2010); and (ii) the torus structure is not fully impermeable, meaning that air bubbles may pass through tiny pores (torus capillary-seeding hypothesis; Jansen *et al.*, 2012). According to recent studies, the first is more likely in most conifer families, while the second may be an additional mechanism in Pinaceae (Jansen *et al.*, 2012). Additional anatomical observations of bordered pits in a broader range of conifers are believed to provide further details about how embolism formation occurs in gymnosperms.

Conifer tracheids are involved not only in water transport but also in mechanical support of the plant. The length of a tracheid, its diameter, and the thickness of its wall are factors contributing to mechanical strength. Based on measurements and theoretical estimations, cavitation resistance is correlated to the ‘thickness to span’ ratio of tracheids (Hacke *et al.*, 2001; Sperry *et al*., 2006; Domec *et al.*, 2009; Arbellay *et al.*, 2012). Higher absolute resistance to cavitation is associated with lower negative sap pressure and requires stronger tracheids with a higher thickness to span ratio to resist to mechanical stress. An increase in the thickness to span ratio, probably due to reduced lumen diameter (Pittermann *et al.*, 2006*b*; Sperry *et al*., 2006), thus also enhances the resistance to water stress. The physiological consequences of this trade-off between hydraulic safety and mechanical strength has received considerable attention (Baas *et al.*, 2004; Domec *et al*., 2006, 2009; Pittermann *et al*., 2006*a*, *b*, 2011; Choat *et al.*, 2007). Therefore, conduit structure is potentially constrained by safety considerations (Sperry *et al.*, 2008).

In this study, the linkage between xylem anatomy and resistance to drought-induced cavitation was assessed using a large database of 115 conifer species. By broadly sampling across conifer phylogeny and four major terrestrial biomes, the objectives were (i) to better understand how xylem anatomical properties associated with air-seeding influence the mechanical strength of the xylem; and (ii) to assess the evolutionary trends of xylem anatomy in the selected taxa. It was hypothesized (i) that the anatomy and functional properties of pit pairs strongly influence cavitation resistance in conifer species—more specifically it was believed that the valve effect (product of torus–aperture overlap and margo flexibility) plays a major role in cavitation resistance; and (ii) that increased cavitation resistance is associated with higher mechanical strength.

## Materials and methods

### Plant material

Anatomical observations and cavitation measurements were conducted on 60 conifer species. Additional data were retrieved from Delzon *et al.* (2010) and Jansen *et al.* (2012) (40 and 15 species, respectively) and completed by measuring novel characteristics. A total of 115 species, including seven families and 45 genera (see Table 1, and Supplementary Table S1 available at *JXB* online), were used to test the relationship between cavitation resistance and anatomical traits. All anatomical observations have been carried out on material that had previously been used for measuring cavitation resistance. These observations were limited to one individual for most species and, in cases where several samples were available, the sample that was closest to the average *P*_50_ value; that is, the xylem pressure inducing 50% loss of hydraulic conductance, was selected. Although the intraspecific variability in pit anatomy can be considerable between organs (roots showed dramatic differences in conduit and pit anatomical properties compared with branches; Domec *et al.*, 2008; Jansen *et al.*, 2009; Schoonmaker *et al.*, 2010), here it was assumed that intraspecific variation was smaller than interspecific variation (Matzner *et al.*, 2001; Maherali *et al.*, 2004; Martinez-Vilalta *et al*., 2009). Moreover, to avoid any additional intraorgan variability, only anatomical features in the xylem of young branches were measured.

**Table 1.** Taxonomic diversity of conifers and species studied (following Farjon, 2008)

Family	Genera	Genera sampled	Species	Species sampled
Araucariaceae	3	3	41	9
Cephalotaxaeae	1	1	11	3
Cupressaceae	30	25	133	43
Pinaceae	11	7	228	35
Podocarpaceae	19	14	186	19
Sciadopityaceae	1	1	1	1
Taxaceae	5	2	23	5
Total	70	53	627	115

### Microscope techniques

#### Light microscopy 

Four to five transverse sections were cut for each sample with a sliding microtome, stained with safranin (1%), and fixed on a microscope slide. Sections were observed with a light microscope (DM2500, Leica, Germany) at the University of Bordeaux. Five photos per section were taken with a digital camera (DFC290, Leica, Germany), and analysed with WinCell (Regent Inst., Canada).

#### Scanning electron microscopy (SEM) 

SEM observations were conducted at Ulm University with a Hitachi cold-field emission scanning electron microscope and with a benchtop scanning electron microscope at the University of Bordeaux (PhenomG2 pro; FEI, The Netherlands). For the Hitachi SEM, thin (1 μm) radial sections were cut in different parts of the stem, air-dried, coated with platinum using a sputter coater (Emitech Ltd; Ashford, UK) for 2min at 10 mA, and observed under 2kV. For the benchtop SEM, samples of 5–8mm long were cut with a razor blade in a radial direction. After drying for 24h in an oven at 70 °C, the samples were fixed on stubs, coated with gold using a sputter coater (108 Auto; Cressington, UK) for 40 s at 20 mA, and observed under 5kV.

#### Transmission electron microscopy (TEM) 

A transmission electron microscope was used to obtain ultrastructural details of pit membranes for 33 species. One stem per species was cut into 1mm^3^ blocks and dehydrated in an ethanol series. The ethanol was gradually replaced with LR White resin (London Resin Co., Reading, UK) over several days (for more details, see Jansen *et al.*, 2012). Then, transverse ultrathin (between 60nm and 90nm) sections were cut using a diamond knife and collected on 100 mesh copper grids. The ultrathin sections were manually stained with uranyl acetate and lead citrate. Observations were carried out with a JEOL JEM-1210 transmission electron microscope (Jeol, Tokyo, Japan) at 80kV accelerating voltage, and digital images were taken using a MegaView III camera (Soft Imaging System, Münster, Germany).

### Xylem anatomical features

**Fig. 1.**
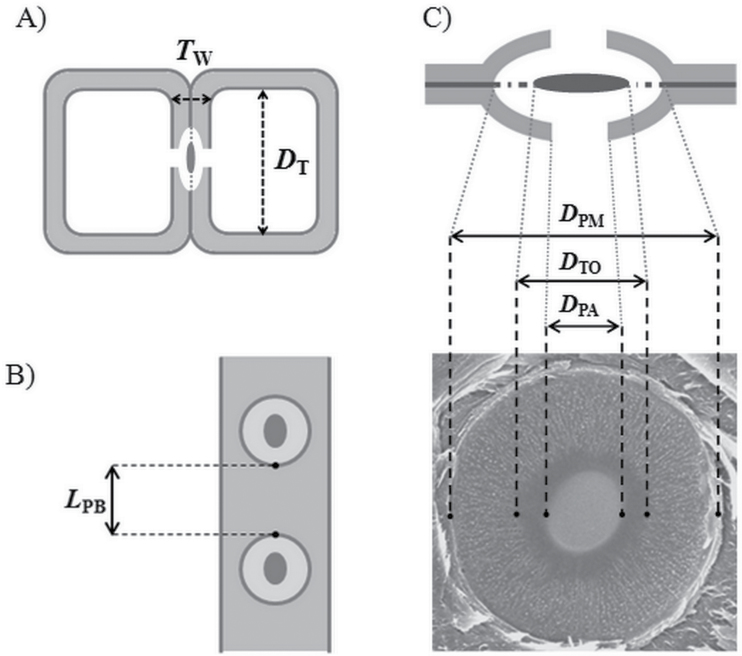
Tracheid and pit membrane structure. (A) Transverse view of two adjacent tracheids. *T*_W_, double wall thickness; *D*_T_, tracheid lumen diameter. (B) Radial view of a pitted wall. *L*_PB_, distance between two adjacent pit borders. (C) Transverse (top) and radial (bottom) view of a bordered pit membrane. *D*_PM_, pit membrane diameter; *D*_TO_, torus diameter; *D*_PA_, pit aperture diameter.

**Fig. 2.**
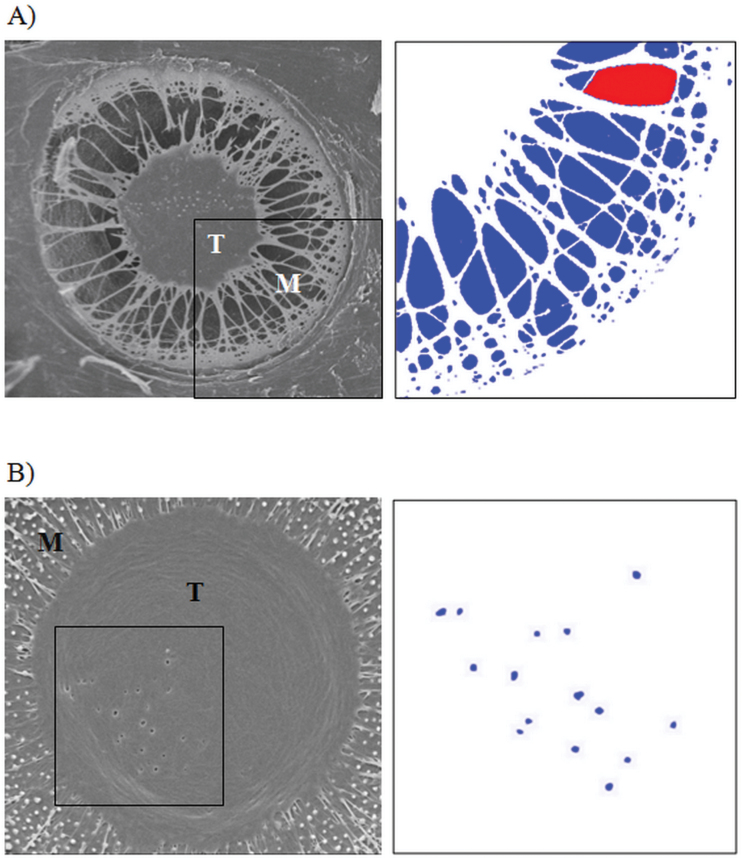
Pit membrane porosity. T, torus; M, margo. (A) Radial view of a pit membrane with a porous margo. ImageJ software was used to measure the diameter of the largest pore in the margo (*D*_MPmax_; red pores) and the average of margo pore diameter (*D*_MP_; average of blue and red pores). (B) Radial view of a pit membrane with punctured torus. ImageJ software was used to estimate the average diameter of the pores in a torus (*D*_TP_).

All anatomical features related to tracheids and bordered pits (see Table 2) were based on earlywood, which is responsible for most of the hydraulic conductance (Domec and Gartner, 2002). Based on light microscopy images (×200 magnification), the tracheid lumen diameter (*D*_T_; the simple average of the equivalent circle diameter) and the thickness of the double wall between neighbouring conduits (*T*_W_) were measured. Pit membrane diameter (*D*_PM_), aperture diameter (*D*_PA_), and torus diameter (*D*_TO_) were measured using SEM (Fig. 1). The distance between two pit borders (*L*_PB_; Fig. 1), the number of margo pores (*N*_MP_), the mean and maximum diameter of margo pores (*D*_MP_, *D*_MPmax_), and the mean diameter of pores in the torus (*D*_TP_) (Fig. 2) were measured using SEM images. Because the *L*_PB_ parameter varies depending on the distribution of pits in tracheids, a constant distribution of pits along the entire length of a tracheid was assumed for these measurements. Average values for all anatomical features were determined based on a minimum of 20 measurements. All anatomical measurements were conducted using ImageJ freeware (W.S. Rasband, ImageJ, US National Institutes of Health, Bethesda, MD, USA, http://imagej.nih.gov/ij/, 1997–2012).

**Table 2.** Anatomical traits and functional properties with reference to their acronym, definition, microscope technique units, and number of measurements

Acronym	Definition	Technique	Unit	Minimum number of measurements
Anatomical features				
*D*_T_	Tracheid lumen diameter: the simple average of the equivalent circle diameter	LM or TEM	μm	30
*D*_MP_	Margo pore diameter	SEM	nm	50
*D*_MPmax_	Maximum margo pore diameter	SEM	nm	50
*D*_PA_	Pit aperture diameter (horizontal diameter at its widest point)	SEM or TEM	μm	20
*D*_PM_	Pit membrane diameter (horizontal diameter at its widest point)	SEM or TEM	μm	20
*D*_TO_	Torus diameter (horizontal diameter at its widest point)	SEM or TEM	μm	20
*D*_TP_	Torus pore diameter	SEM	nm	20
*N*_MP_	Number of pores in a margo	SEM	–	–
*L*_PB_	Distance between pit adjacent borders	SEM	μm	20
*T*_TW_	Tracheid wall thickness measured as the double wall between two adjacent tracheids	LM or TEM	μm	30
Functional traits				
*D*_H_	Hydraulic diameter=Ʃ*D*_T_^5^/Ʃ*D*_T_^4^	–	μm	–
*F*	Flexibility of the margo=(*D*_PM_–*D*_TO_)/*D*_TO_	–	–	–
*L*_E_	Ligament efficiency	–	–	–
*O*	Torus overlap=(*D*_TO_–*D*_PA_)/*D*_TO_	–	–	–
*P*_WI_	Wall implosion pressure	–	MPa	–
*P*_MC_	Margo capillary seeding pressure	–	MPa	–
*P*_RS_	Rupture stretching pressure	–	MPa	–
*P*_TC_	Torus capillary-seeding pressure	–	MPa	–
*P*_TD_	Torus deflection pressure	–	MPa	–
*R*_PA_	Pit aperture resistance	–	MPa.s m^–3^	–
*R*_MP_	Margo pore resistance	–	MPa.s m^–3^	–
*R*_P_	Total pit resistivity	–	MPa.s m^–3^	–
*T*_W_ *D*_T_^–^	Thickness to span ratio	–	MPa.s m^–3^	–
*V*_EF_	Valve effect=*O*×*F*	–	–	–

### Xylem anatomical properties

The following functional properties of pit membranes and tracheids (Table 2) were estimated from anatomical measurements to investigate micro-morphological variation in relation to embolism formation.

#### Seal capillary seeding 

The margo flexibility [*F*=(*D*_PM_–*D*_TO_)/*D*_TO_], the torus overlap [*O*=(*D*_TO_–*D*_PA_)/*D*_TO_], and the valve effect (*V*_EF_=*F*×*O*) were estimated following Delzon *et al.* (2010). *D*_PM_ is the pit membrane diameter, *D*_PA_ the pit aperture diameter, and *D*_TO_ is the torus diameter.

#### Margo and torus capillary seeding pressure 

The pressure difference between two adjacent tracheids required to break the air–water meniscus in the margo was calculated following the Young–Laplace equation:

$$P_{\text{MC}}=–[\text{4cos}\tau \left(\alpha \right)]/D_{\text{MP}}$$

where *τ* (0.0728 N m^–1^ at 20 °C) is the surface tension of water, α is the contact angle between the microfibrils and the meniscus (assumed equal to 0°), and *D*_MP_ is the average diameter of the margo pores.

The pressure difference required to break the air–water meniscus in the torus when it is already sealed against the pit aperture was calculated as:

$$P_{\text{TC}}=–[\text{4cos}\tau \left(\alpha \right)]/D_{\text{TP}}$$

where *D*_TP_ is the mean diameter of a pore in the torus.

#### Torus deflection 

The pressure difference required to aspirate the pit membrane onto the pit border was estimated according to Domec *et al.* (2006):

$$P_{\text{TD}}=–\left(\text{2}N\text{MOE}e_{\text{A}}\right)]/(\pi rD_{\text{PM}})$$

where *N* is the number of margo strands, which was assigned to the average value of 55 and 200 strands per pit (Domec *et al.*, 2006). MOE is the modulus of elasticity of the strands (taken at 3.5 GPa; Domec *et al.*, 2006) and *e*_A_ is the margo spoke strain at aspiration (*e*_A_=0.03*D*_PM_/*D*_PM_–*D*_TO_).

#### Rupture stretching 

The pressure difference needed to break strands of the margo was estimated following Domec *et al.* (2006):

$$P_{\text{RS}}=–\text{2}JN\left\{\left(D_{\text{PM}}/\text{2}r\right)/\pi \left[D_{\text{TO}}/\text{2}+{\left(D_{\text{PA}}/\text{2}\right)}^{\text{2}}\right]\right\}–P_{\text{TD}}$$

where *J* is the tension of a strand between the aspirated position and stretched position when the torus goes through the pit aperture and no longer covers the whole aperture [*J*=0.0147(*e*_S_–*e*_A_)MOE] and *e*_S_ is the margo spoke strain in the stretched position [*e*_S_=(*D*_TO_–*D*_PA_+0.03*D*_PM_)/(*D*_PM_–*D*_TO_)].

#### Pit hydraulic resistance 

The hydraulic trade-off associated with cavitation resistance was quantified by calculating the pit aperture resistivity (*R*_PA_), margo resistivity (*R*_M_), and total pit resistance (*R*_P_) following Hacke *et al.* (2004) and Pittermann *et al.* (2010):

$$R_{\text{PA}}=[\text{128}T_{\text{PA}}\upsilon /{\left(\pi D_{\text{PA}}\right)}^{\text{4}}+\text{24}\upsilon /D_{\text{PA}}{}^{\text{3}}]$$

where υ is the viscosity of the water (0.001 Pa.s at 20°C) and *T*_PA_ is the thickness of a single pit border calculated from the double wall thickness (*T*_PA_=81% of *T*_W_; Domec *et al.*, 2008).

$$R_{\text{M}}=\left[\text{24}\upsilon /{\left(N_{\text{MP}}D_{\text{MP}}\right)}^{\text{3}}\right]f\left(h\right)$$

where *N*_MP_ is the number of pores in the margo and *f*(*h*) is the function of *h*, the proportion of the margo occupied by pores [*h*=*N*_MP_π(*N*_MP_/2)^2^/π(*D*_PM_/2)^2^]. The total pit resistance *R*_P_ equals the sum of *R*_PA_ and *R*_M_ (*R*_P_ *=R*_PA_+*R*_M_).

The thickness to span ratio was also estimated as it reflects the tracheid contribution to conductance. It corresponds to *T*_W_/*D*_T_, where *T*_W_ is the double wall thickness and *D*_T_ the tracheid lumen diameter (the simple average of the equivalent circle diameter).

#### Xylem failure by theoretical conduit implosion 

The conduit implosion pressure was defined as the pressure difference across the tracheid wall required to cause bending stress to exceed the wall strength. It was estimated using methods described by Domec *et al.* (2008):

$$P_{\text{WI}}=(\omega /\beta ){\left(T_{\text{W}}/D_{\text{T}}\right)}^{\text{2}}L_{\text{E}}\left(I_{\text{H}}/I_{\text{S}}\right)$$

where ω is the strength of the wall material assumed to be 80MPa (Hacke *et al.*, 2001), and β is a coefficient taken as 0.25. The moment ratio, *I*_H_/*I*_S_, represents the ratio of the second moment of area of a wall with pit chamber (*I*_H_) to that of a solid wall with no pit chamber present (*I*_S_). Hacke *et al.* (2004) showed that *I*_H_/*I*_S_ does not change with air-seeding pressure, and is on average ~0.95 in conifers. The ligament efficiency [*L*_E_=1–*D*_PA_/(*D*_PM_+*L*_PE_)] quantifies the spatial distribution of the pit aperture in the wall.

### Cavitation resistance

Cavitation resistance data were retrieved from Delzon *et al.* (2010; unpublished data). Data analyses were carried out for several cavitation traits, *P*_50_ (xylem pressure inducing 50% loss of hydraulic conductance), *S* (slope of the vulnerability curves) (Pammenter and Vander Willigen, 1998), *P*_12_ (xylem air entry), and *P*_88_ (xylem pressure at which 88% of conductivity is lost) (for more details, see Domec and Gartner, 2001). As similar results were found for all cavitation parameters, it was decided to present the relationship between anatomical traits and *P*_50_ for simplicity. However, xylem air entry pressure (*P*_12_) was also used to test whether cavitation occurs before or after torus deflection.

### Data analysis

Cross-species correlations between xylem anatomical traits and cavitation resistance were tested with a Pearson correlation coefficient (*r*) and a Spearman correlation coefficient (*s*) for non-linear data. In addition, the species distribution in the four major biomes was retrieved from Delzon *et al*. (unpublished data), and variations of anatomical traits among biomes were assessed using a one-way analysis of variance (ANOVA). Data and statistical analyses were conducted using the SAS software (version 9.3 SAS Institute, Cary, NC, USA).

Phylogeny can induce bias when testing for correlations in pairs of traits within a group of species. Because of shared evolutionary history between related species, the assumption of independence of classical statistical tests and correlations is disregarded (Felsenstein, 1985; Harvey and Pagel, 1991; Garland *et al.*, 1992). The phylogenetically independent contrast method (or PIC; Felsenstein, 1985) is a common workaround for this issue: differences (or contrasts) in trait values are computed for each pair of species and each node of the phylogeny; these are statistically independent, and represent the evolutionary divergences in traits at each node (for more detailed information, see Felsenstein, 1985; Garland *et al.*, 1992). Furthermore, the PIC method is used to test for correlated evolution between traits: a significant positive trend (i.e. PICs for trait A are positively correlated with PICs for trait B) means that for each node of the phylogeny a change in the value of trait A is associated with the evolution of trait B. Phylogenetically independent contrasts analyses (PICs; Felsenstein, 1985) were run in R (R Development Core Team, 2008) using the ‘ape’ package (Paradis *et al.*, 2004).

This method requires knowledge of the phylogeny of the studied taxa with branch lengths. To this end, DNA sequences for three generally available genes (chloroplast genes *rbc*L and *mat*K, and nuclear gene *phy*P) were retrieved from GenBank (Benson *et al.*, 2010) and aligned using PHLAWD (PHyLogeny Assembly With Databases; Smith *et al.*, 2009; http://code.google.com/p/phlawd). Alignements were visually checked and trimmed in MEGA 5.0 (Tamura *et al.*, 2011). Maximum Likelihood (ML) phylogenetic analyses were run in RAxML (version 7.0.3; Stamatakis, 2006). A separate GTR+CAT rate model was assigned to each partition, and each search was conducted 100 times and the best maximum likelihood (ML) tree was retained.

## Results

Inclusion of phylogenetic information was useful to identify adaptive relationships between anatomical properties and cavitation resistance. Below both cross-species correlations between variables analysed with Pearson or Spearman correlation (*r* or *s*) coefficients and phylogenetically independent contrasts (PICs) are presented.

### Anatomical properties

Across species, cavitation resistance (*P*_50_) was negatively correlated with pit aperture diameter (*D*_PA_; Table 3) showing that species with narrower pit apertures are more resistant to cavitation. However, these relationships were not supported by correlations using PICs (Table 3). No relationship was found between cavitation resistance and other anatomical traits such as tracheid lumen diameter (*D*_T_), torus diameter (*D*_TO_), or pit membrane diameter (*D*_PM_). The same results were found for correlations with *P*_12_, *P*_88_, and the slope of vulnerability curves (Supplementary Table S2 available at *JXB* online).

**Table 3.** Pearson (*r*) and Spearman correlation (*s*), and phylogenetically independent contrast correlations (PICs) for relationships between anatomical and functional traits with cavitation r**e**sistance (*P*_50_) in conifersThe number of species measured is mentioned for each parameter.

	Correlation with *P*_50_			PIC		*n*
	*r*	*s*	*P*-value	PIC	*P*-value	
Pit membrane properties						
*D*_MP_	0.21		0.19			38
*D*_MPmax_	0.05		0.71			38
***D***_**PA**_		**–0.30**	**0.002**	–0.13	0.23	**97**
*D*_PM_	0.12		0.21	0.18	0.07	96
*D*_TO_	0.06		0.54			88
*F*		–0.04	0.7	0.02	0.82	88
*N*_MP_	0.22		0.14			42
***O***		**0.46**	**<0.0001**	**0.24**	**0.03**	**87**
*P*_MC_	0.1		0.94			38
***P***_**RS**_	**–0.43**		**0.0002**			**65**
***P***_**TC**_	**0.52**		**0.046**			16
*P*_TD_	0.20		0.10			66
***V***_**EF**_		**0.52**	**<0.0001**	**0.30**	**0.006**	**87**
Mechanical features						
*D*_T_	- 0.17		0.12			72
***D***_**H**_		**–0.31**	**0.007**	-0.06	0.59	**72**
*T*_W_		0.15	0.17	0.22	0.007	81
Mechanical properties						
***P***_**WI**_		**–0.51**	**0.0001**	**0.49**	**<0.0001**	**63**
***T***_**W**_ ***D***_**T**_^**–1**^		**0.41**	**0.0003**	**0.30**	**0.01**	**73**
Pit membrane resistance						
***R***_**PA**_		**0.30**	**0.01**	**0.23**	**0.05**	**73**
*R*_MP_		0.09	0.57	**0.37**	**0.03**	38
***R***_**P**_		**0.47**	**0.005**	-0.09	0.60	**33**

Bold values indicate significant correlations at *P*<0.05.

### Pit membrane functional properties

**Fig. 3.**
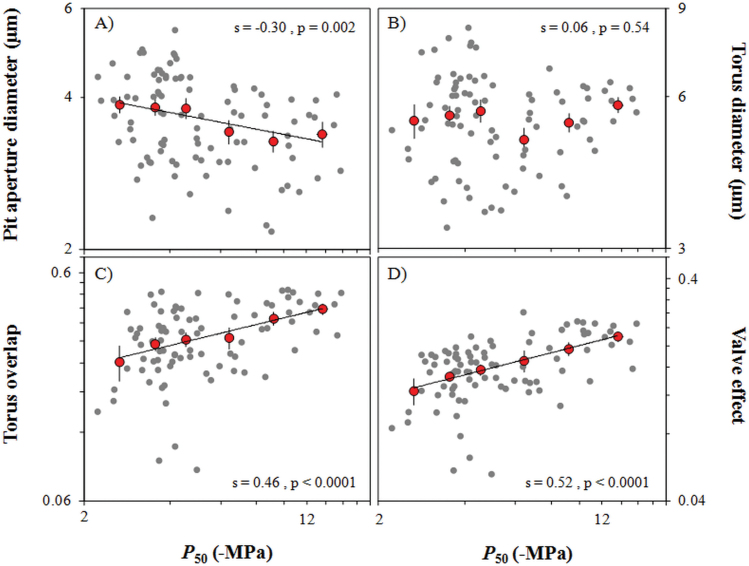
Relationship between cavitation resistance (*P*_50_) and anatomical traits [pit aperture diameter (A), torus diameter (B)] and functional properties [torus overlap (C), valve effect (D)] of pit membrane in conifers. Red circles are binned into ranges of *P*_50_ and plotted in log scale. Raw data (mean values per species) are shown as small grey points behind binned data. Linear regressions shown are based on raw data and indicate when the correlation is significant.

*P*_50_ was significantly correlated with functional properties of the bordered pit such as torus–aperture overlap (*O*) and valve effect (*V*_EF_; product of torus overlap and margo flexibility) (Table 3, Fig. 3). This relationship was also supported by the PIC analyses, suggesting a correlative evolution between cavitation resistance and pit membrane functional properties. Increasing valve effect (*V*_EF_) increased cavitation resistance. Variation in valve effect was mainly due to change in torus–aperture overlap as margo flexibility varied weakly among species. Following cross-species correlations, pit aperture diameter (*D*_PA_) contributed more to torus–aperture overlap than to torus diameter (*D*_TO_) (Fig. 3). However, the PIC analyses did not confirm this trend (Table 3).

Based on interspecific analyses, rupture stretching pressure (*P*_RS_) was highly correlated to *P*_50_ (Table 3). Margo strength increased with increasing cavitation resistance. However, *P*_RS_ was always much lower than *P*_50_ (*P*_RS_= –4.67 to –71.31MPa; *P*_50_= –2.23 to –15.79MPa; Supplementary Fig. S1 available at *JXB* online), suggesting that cavitation took place before mechanical rupture of the margo strands. For all species, torus aspiration occurred at a relatively high xylem pressure (close to 0MPa, *P*_TD_= –0.03 to –0.33MPa; Supplementary Fig. S1 available at *JXB* online) as compared with the xylem air entry pressure (*P*_12_= –0.91 to –12.66MPa; Supplementary Fig. S1 available at *JXB* online). Moreover, the pressure difference inducing margo capillary rupture (*P*_MC_) was lower (more negative) than the pressure difference required to deflect the torus towards the pit aperture (*P*_TD_) (*P*_MC_= –0.10 to –0.75MPa; Supplementary Fig. S1 available at *JXB* online). This means that torus deflection occurs before an air–water meniscus breaks through the margo of a pit membrane. Cavitation resistance was positively correlated with total pit resistivity (*R*_P_) and pit aperture resistivity (*R*_PA;_ Table 3; Fig. 5A, C) but not with margo resistivity (*R*_M_; Table 3; Fig. 5B), suggesting that the margo pores are not involved in cavitation resistance in conifers. However, these trends were not confirmed by the PIC analysis (Table 3). Concerning the 70 species tested for the torus capillary-seeding hypothesis, only 16 species were found with punctured tori, mostly belonging to the Pinaceae family. The size of the pores varied from 12nm to 144nm. Only a weak but significant correlation was found between cavitation resistance and torus capillary-seeding pressure (*P*_TC_, *s*=0.52, *P*=0.046; Supplementary Fig. S2 available at *JXB* online). The air-seeding pressure based on the size of plasmodesmatal pores in tori was of the same order of magnitude as the *P*_50_ values.

### Mechanical properties

**Fig. 4.**
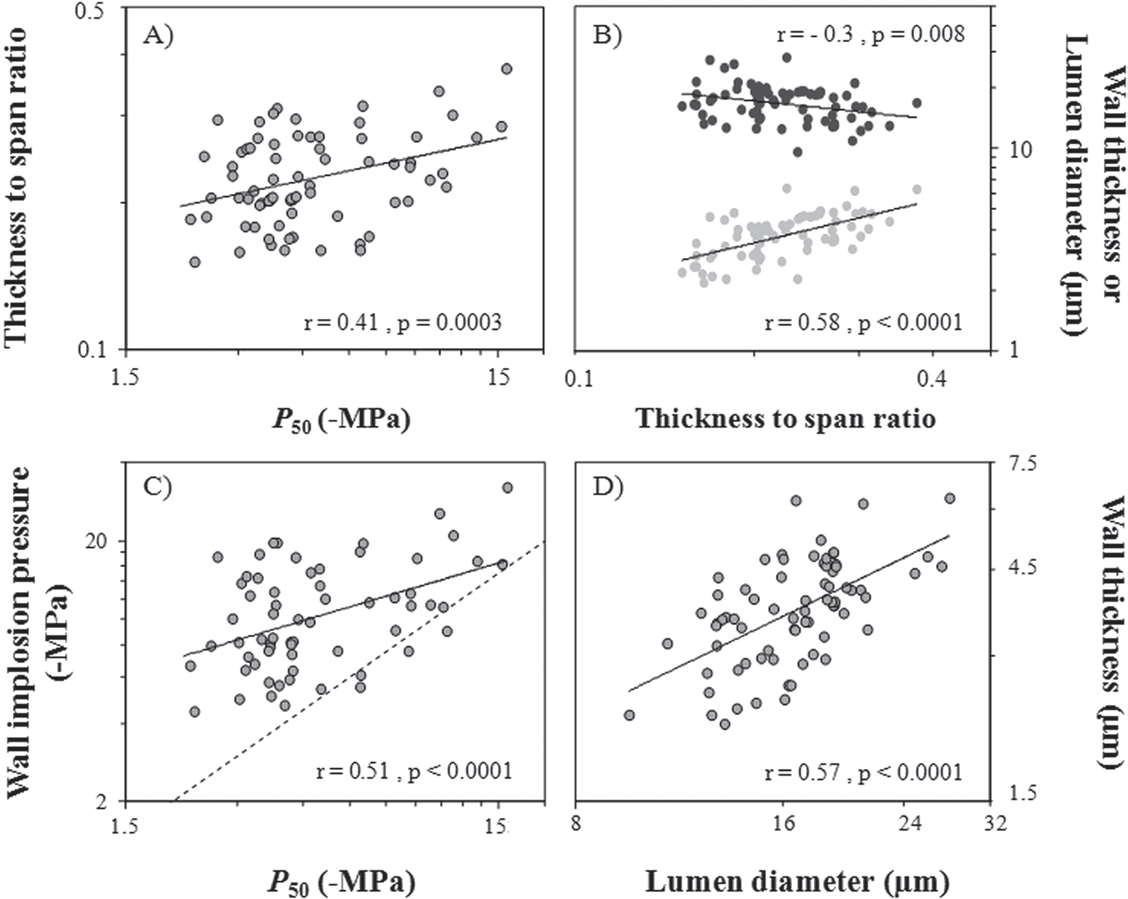
Relationship between (A) thickness to span ratio and cavitation resistance (*P*_50_); (B) wall thickness (grey circles), lumen diameter (black circles), and thickness to span ratio; (C) wall implosion pressure and cavitation resistance (*P*_50_); and (D) wall thickness and lumen diameter. Regression lines are indicated when the correlation is significant.

Spearman correlations and PIC analyses showed a positive correlation between the thickness to span ratio (*T*_W_ *D*_T_^–1^) and cavitation resistance (*P*_50_; Fig. 4A; Table 3). Variation in *T*_W_ *D*_T_^–1^ across conifers was mainly determined by changes in double cell-wall thickness (*T*_W;_ *s*=0.60, PIC=0.57) rather than a change in tracheid lumen diameter (*D*_T_; Fig. 4B). Moreover, tracheid lumen diameter and wall thickness were positively correlated (*s*=0.65, PIC=0.52; Fig. 4C), meaning that wall thickness and tracheid lumen diameter varied in the same way when total tracheid size changed. Wall implosion pressure (*P*_WI_) was also positively correlated with cavitation resistance (Table 3; Fig. 4C). For most of the species, *P*_WI_ was always more negative than *P*_50_ (*P*_WI_= – 4.69 to – 32.14MPa), suggesting that conduit implosion does not occur before cavitation. Comparing the data plot in Fig. 4C with the 1:1 line, the difference between *P*_WI_ and *P*_50_ decreased from vulnerable species (low absolute *P*_50_ values) to resistant species (high absolute *P*_50_ values). The PIC analyses suggest that traits related to cavitation resistance and mechanical strength have evolved jointly.

**Fig. 5.**
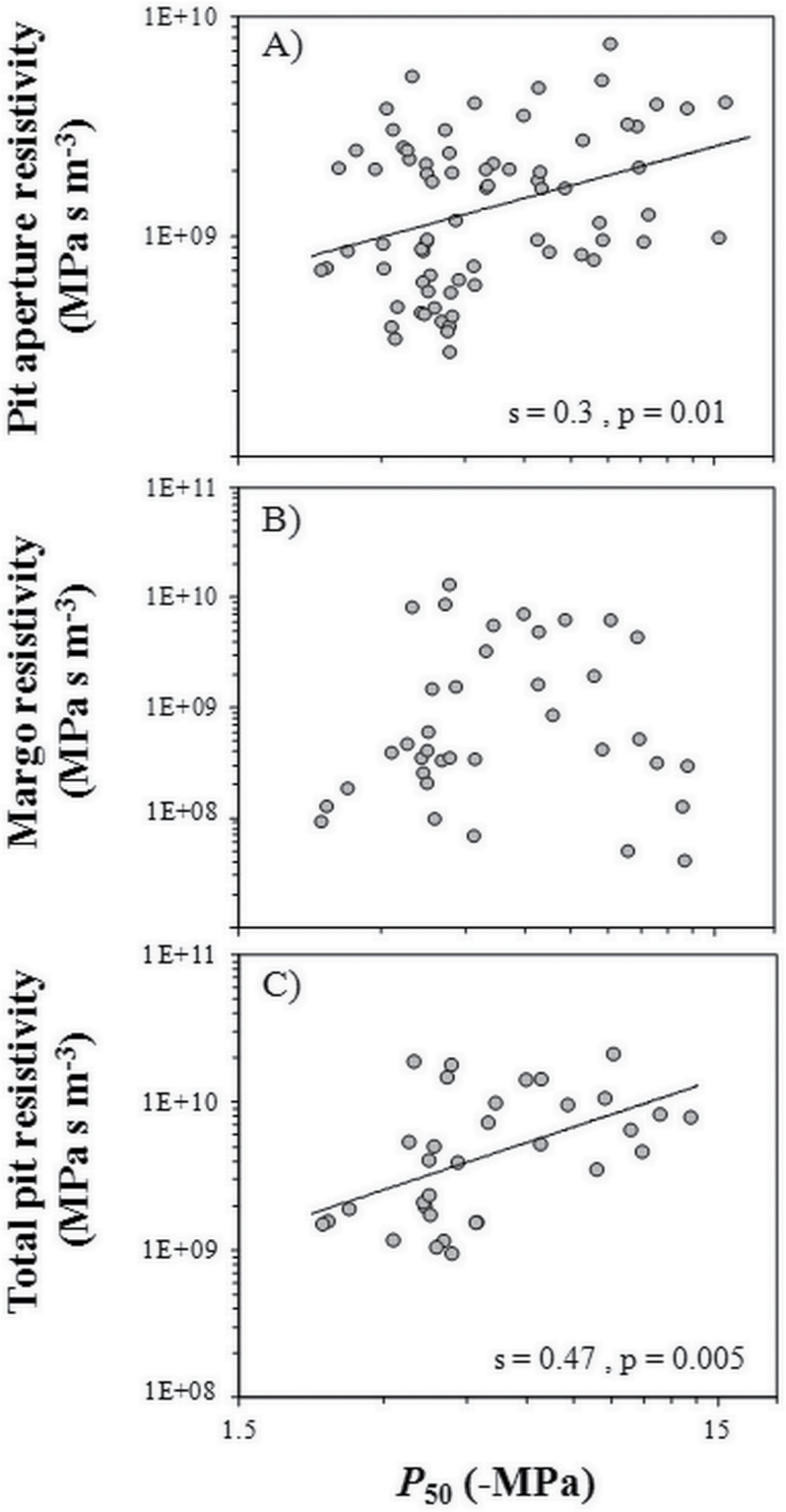
Relationship between pit aperture resistance (A), margo resistance (B), and total pit resistance (C) and cavitation resistance (*P*_50_). The regression line is indicated when the correlation is significant.

### Species distribution

Significant differences in xylem anatomy and hydraulic properties were found between the four biomes. In general, torus–aperture overlap, valve effect, and thickness to span ratio were significantly higher for the Mediterranean biome than the other biomes (Table 5). Species from Mediterranean regions showed a 2-fold higher cavitation resistance (more negative *P*_50_) than in the other biomes (Table. 5).

**Table 4.** Ratio of species with punctured tori per total number of species studied with reference to their taxonomic family

Family	Punctured torus species/total species^*a*^
Araucariaceae	–
Cephalotaxaeae	1/3
Cupressaceae	3/32
Pinaceae	15/17
Podocarpaceae	0/15
Sciadopityaceae	0/1
Taxaceae	0/4
Total	19/72

^*a*^ Species studied for the torus capillary-seeding hypothesis.

**Table 5.** Variation in anatomical and hydraulic traits among biomesMean values of *P*_50_, torus–aperture overlap (*O*), valve effect (*V*_EF_), and thickness to span ratio (*T*_W_/*D*_T_) for the four main biomes represented in this study.

	Torus–aperture overlap	Valve effect	Thickness to span ratio	*P*_50_
Mediterranean	0.41±0.02 a	0.21±0.008 a	0.25±0.01 a	7.93±0.1 a
Tropical	0.29±0.03 b	0.16±0.01 b	0.23±0.01 ab	4.23±0.2 b
Temperate	0.28±0.01 b	0.14±0.006 b	0.22±0.008 ab	3.87±0.34 b
Boreal	0.32±0.03 b	0.15±0.01 b	0.19±0.01 b	3.39±0.62 b
*P*-value	<0.0001	<0.0001	0.01	<0.0001

A *P*-value <0.05 indicates a significant difference between biomes, and the letters (a, b) indicate to what extent the biomes differ from each other.

## Discussion

### Pit anatomy and cavitation resistance

Across the 115 species studied, bordered pit properties of earlywood tracheids (torus–aperture overlap and valve effect) are the best proxy to explain the variability of cavitation resistance: species resistant to cavitation have a high valve effect (corresponding to both torus–aperture overlap and membrane flexibility). This is consistent with the seal capillary-seeding hypothesis (Delzon *et al.*, 2010). Regarding the flexibility of the margo, previous studies suggested that this feature could play an important role in the process of cavitation. First, Hacke *et al.* (2004) mentioned that margo flexibility could allow the torus to be pulled out through the pit aperture, so that species that are vulnerable to cavitation should have a more flexible margo. In contrast, Delzon *et al.* (2010) reported that a high margo flexibility may facilitate the torus to move toward the pit border and improve the seal between the torus and the pit aperture. The present study showed that most of the valve effect efficiency is due to variation in the torus–aperture overlap, while the flexibility of the margo does not seem to play a substantial role. Furthermore, the results confirm that torus capillary-seeding may provide an additional air-seeding mechanism in Pinaceae. While this must be interpreted with caution because SEM observations do not allow pores that completely pass through the torus to be distinguished from those that are limited to the surface of the torus, Jansen *et al.* (2012) showed that at least a few pores in each species with punctured tori completely pass through the torus. Punctured tori are also observed in some members of Cupressaceae and Cephalotaxaceae (see Table 4, and Jansen *et al.*, 2012). Plasmodesmata need to be associated with early developmental stages of the torus in combination with a lack of matrix removal from the torus by autolytic enzymes during cell hydrolysis (Murmanis and Sachs, 1969; Dute, 1994; Dute *et al.*, 2008). Thus, the taxonomic limitation of punctured tori to these families is assumed to reflect developmental differences in torus ontogeny. However, because of the lack of a distinct torus in Araucariaceae (Bauch *et al.*, 1972), no comment can be made on the mechanism of air-seeding for this family. Nevertheless, in terms of cavitation resistance, Araucariaceae are highly vulnerable to air-seeding (*P*_50_= –2.02 to –3.3MPa). Pittermann *et al.* (2010) showed that the more cavitation resistant a species is the more pronounced is the torus–margo difference.

In this study, two additional mechanisms of air-seeding were also investigated, and they were excluded in agreement with Cochard *et al.* (2009). The pressure difference needed to break the margo (*P*_RS_) was more negative than *P*_50_, regardless of the number of margo strands (Supplementary Fig. S1 available at *JXB* online). Therefore, cavitation was not due to breaking of the margo strands but to capillary rupture of the air–sap meniscus (Cochard *et al.*, 2009). Furthermore, the results demonstrate that cavitation does not occur at the pores in the margo, but when the torus becomes aspirated against the pit border and seals the pit aperture (Petty, 1972; Sperry and Tyree, 1990; Hacke *et al.*, 2004; Domec *et al.*, 2006; Delzon *et al.*, 2010).

### Mechanical properties

The present data indicated a strong trade-off between hydraulic and mechanical safety with a significant evolutionary association between increasing cavitation resistance and increasing thickness to span ratio of tracheids. As in previous studies (Sperry *et al*., 2006; Pittermann *et al.*, 2005), the present results show that species resistant to cavitation have thicker tracheid walls relative to lumen area. While Pittermann *et al.* (2006*b*) and Sperry *et al*. (2006) concluded that variation in the thickness to span ratio was determined by lumen diameter rather than cell wall thickness, the present study demonstrated the opposite: the wall thickness but not the lumen diameter appears to be responsible for the trade-off between hydraulic safety and mechanical strength. A mechanical constraint on xylem anatomy could explain this relationship. Higher cavitation resistance is associated with lower negative sap pressure (Maherali *et al.*, 2004; Choat *et al.*, 2012), which requires tracheids with a higher thickness to span ratio to resist mechanical stresses. Cavitation resistance might therefore be indirectly linked to thickness to span ratio. From a functional perspective, thicker walls in relation to lumen area do not improve drought-related cavitation resistance as this phenomenon occurs at the bordered pit level. Taking into account that there is certainly a carbon cost limitation in building up tracheid walls, Sperry *et al*. (2006) suggested that walls are close to their maximum thickness. Thereby, conifer trees can only compete for higher mechanical strength by narrowing their tracheid lumina, while levels of ray and axial parenchyma remain typically low. However, our study showed that as cell diameter increased, cell-wall thickness varied proportionally much more than lumen diameter. This suggests that there could be a minimum lumen diameter threshold that maintains a minimum level of hydraulic conductance.

The results confirm that hydraulic failure by implosion is unlikely in lignified tracheids of conifers. Conduit implosion of xylem has been observed in stems of a few Pinaceae, but only in tracheids with severe reduction of lignification in their secondary walls (Barnett, 1976; Donaldson, 2002). Indeed, some degree of lignification is required to allow normal xylem function and water conduction. The data show that the pressure needed to cause conduit implosion in lignified tracheids is related to cavitation resistance, but is for most species more negative than *P*_50_. The minimum water potential measured in conifer species is generally less negative than *P*_50_, suggesting that the conduit implosion pressure is unlikely to occur under field conditions (Choat *et al.*, 2012). These results suggest that cavitation always occurs before xylem collapse. According to Domec *et al.* (2009), safety factors for implosion are high compared with air-seeding. One interpretation of such a safety margin is that there has been strong selective pressure to avoid implosion (Pittermann *et al.*, 2006*b*). Even so, evidence of collapse has been observed in needles of Podocarpaceae, but localized in extra-xylary transfusion tracheids (Brodribb and Holbrook, 2005). Dysfunction by collapse seems more likely to occur in these cells than in the xylem because of their parenchymatous origin, irregular shape, large lumen, and high pit density (Aloni *et al.*, 2013).

### Species distribution

Only a few investigations were carried out on the relationship between conifer species distribution and xylem anatomy. Previous studies on hydraulic traits have provided evidence of considerable variations of cavitation resistance between species from contrasting environments, with individuals from xeric regions more resistant than those from mesic regions (Maherali *et al.*, 2004; Choat *et al*., 2005, 2012; Vinya *et al.*, 2013). Swenson and Enquist (2007) and Slik *et al*. (2008) highlighted the existence of geographic variations in wood density with significantly denser wood in dryer habitats. The present study confirmed these trends and, in addition, it was hypothesized that xylem anatomical and, in particular, bordered pit properties would differ between the different biomes, in agreement with the variability of cavitation resistance. The distribution analyses strengthen the conclusion that species growing in arid environments such as the Mediterranean region present the following combination of distinct anatomical features: wide torus–aperture overlap, high valve effect, and large thickness to span ratio. Results from the variation in cavitation resistance in combination with the distribution of species showed a substantial ability of species from xeric habitats to resist drought-induced cavitation and support mechanical strength. However, it does not mean that conifers from Mediterranean habitats are immune to drought stress (Choat *et al.*, 2012).

### Conclusion

Based on both cross-species correlations and PIC analyses, the wide taxonomic sample examined here enabled the demonstration that cavitation is most likely to occur by seal capillary-seeding via the overlap of the torus on the pit aperture, while torus capillary-seeding could provide an additional mechanism in Pinaceae. Testing the consequences of increasing cavitation resistance highlighted an indirect trade-off between hydraulic safety (*P*_50_) and mechanical strength (thickness to span ratio) over a broad range of species. This study illustrates that the torus–aperture overlap and the thickness to span ratio represent the two most useful proxies to estimate cavitation resistance. It was also found that dysfunction by conduit implosion in xylem tracheids is unlikely as the theoretical implosion pressure is unrealistic for most species. Secondly, increased cavitation resistance did not come at a cost of decreased tracheid lumen diameter and should therefore have only a minor impact on hydraulic efficiency. Compared with angiosperms, conifers seem to be able to achieve greater cavitation resistance without considerably sacrificing hydraulic efficiency. This growth strategy could allow conifers to colonize seasonally arid habitats that are subject to freezing-induced embolism formation (Hacke *et al.*, 2005). The bordered pit anatomy of the xylem could slightly affect the ability of the species to resist drought-induced embolism and consequently conifer distribution. Although it is felt that the approach used here of studying few individuals per species is valid when covering a large number of species, further work on the intraspecific and intraindividual variability of conifers would be required to better understand hydraulic trade-offs and functional adaptations to different environments.

## Supplementary data

Supplementary data are available at *JXB* online.

Figure S1. Relationship between (A) cavitation resistance (*P*_50_) and rupture stretching pressure, (B) xylem air entry pressure (*P*_12_) and torus deflection pressure, and (C) margo capillary-seeding pressure and torus deflection pressure.

Figure S2. Relationship between torus capillary-seeding pressure and cavitation resistance (*P*_50_).

Table S1. List of species studied with reference to their taxonomic family, origin, and average cavitation resistance values (*P*_50_).

Table S2. Pearson (*r*) and Spearman correlation (*s*) for relationship between anatomical/functional traits and cavitation traits (*P*_50_, *P*_12_, *P*_88_, and slope) in conifers.

Supplementary Data
