# Supplementary material for: A broad survey of hydraulic and mechanical safety in the xylem of conifers
Source: J Exp Bot. 2014 Jun 10;65(15):4419–31. doi: 10.1093/jxb/eru218 (PMC4112641; doi:10.1093/jxb/eru218)
Supplement: Supplementary Data [file supp_65_15_4419__index.html]

A broad survey of hydraulic and mechanical safety in the xylem of conifers — A broad survey of hydraulic and mechanical safety in the xylem of conifers — Supplementary Data 

# A broad survey of hydraulic and mechanical safety in the xylem of conifers

## Supplementary Data

Data files

**Files in this Data Supplement:**

- Supplementary Data - Supplementary Data
