# Supplementary material for: A broad survey of hydraulic and mechanical safety in the xylem of conifers
Source: J Exp Bot. 2014 Jun 10;65(15):4419–31. doi: 10.1093/jxb/eru218 (PMC4112641; doi:10.1093/jxb/eru218)
Supplement: Supplementary Data [file supp_eru218_jexbot121186_file001.pdf]

A broad survey of hydraulic and mechanical safety in the xylem of conifers.

Pauline S. Bouche[1,2,3], Maximilien Larter[2,3], Jean-Christophe Domec[4,5], Régis Burlett[3], Peter Gasson[6], Steven Jansen[1], and Sylvain Delzon[2,3]

Supplementary tables

Table S1. List of species studied with reference to their taxonomic family, origin and average cavitation resistance values (P50).[a] Material studied in this paper; [b] Material studied by Delzon et al. 2010; [c] Material studied by Jansen et al. 2012.

Table S2. Pearson (*r*) and Spearman correlation (*s*) for relationship between anatomical/ functional traits and cavitation/hydraulic traits (P_{50}, P_{12}, P_{88} and slope) in conifers.

.

Table S1.

Family	Species	Authority	Origin and accesion number	P50 (MPa) ± SE
Araucariaceae	*Agathis atropurpurea* [a]	Hyland	RBG Sydney, 20100069	-2.89 ± 0.27
Araucariaceae	*Agathis australis* [a]	(D.Don) Lindl.	Bedgeburry National Pinetum, 17-0581	-2.13
Araucariaceae	*Agathis microstachya* [a]	J.F.Bailey & C.T.White	RBG Mount Annan, 865566	-2.55 ± 0.07
Araucariaceae	*Agathis robusta* [a]	(C.Moore ex F.Muell.) F.M.Bailey	RBG Mount Annan	-2.90 ± 0.06
Araucariaceae	*Araucaria araucana* [a]	(Molina) K.Koch	Argentina	-3.06 ± 0.81
Araucariaceae	*Araucaria bidwillii* [a]	Hook.	RBG Sydney, 19074	-3.01 ± 0.08
Araucariaceae	*Araucaria cunninghamii* [a]	Aiton ex D.Don	RBG Sydney, 863087	-2.64 ± 0.08
Araucariaceae	*Araucaria heterophylla* [a]	(Salisb.) Franco	RBG Sydney, 15960	-2.96 ± 0.07
Araucariaceae	*Araucaria hunsteinii* [ab]	K.Schum.	RBG Sydney, 902638	-2.43 ± 0.08
Araucariaceae	*Wollemia nobilis* [a]	W.G.Jones, K.D.Hill & J.M.Allen	RBG Sydney	-3.32 ± 0.15
Cephalotaxaceae	*Cephalotaxus fortunei* [ac]	Hook.	RBG Kew, 1969-16466	-7.26 ± 0.48
Cephalotaxaceae	*Cephalotaxus harringtonia* [ac]	(Knight ex J.Forbes) K.Koch	RBG Kew, 1969-16244	-7.21± 0.47
Cephalotaxaceae	*Cephalotaxus wilsoniana* [a]	Hayata	Bedgeburry National Pinetum, 22-0202	-7.92 ± 0.29
Cupressaceae	*Actinostrobus pyramidalis* [b]	Miq.	Clermont-Ferrand, France	-10.72 ± 0.57
Cupressaceae	*Athrotaxis cupressoides* [a]	D.Don	RBG Kew, 2003-2212	-3.16 ± 0.34
Cupressaceae	*Athrotaxis laxifolia* [a]	Hook.	Bedgeburry National Pinetum, 10-671	-2.47 ± 0.19
Cupressaceae	*Austrocedrus chilensis* [a]	(D.Don) Pic.Serm. & Bizzari	Argentina	-4.96 ± 0.19
Cupressaceae	*Callitris columellaris* [ac]	F.Muell.	University of Tasmania, Hobart	-15.79 ± 0.18
Cupressaceae	*Callitris endlicheri* [a]	(Parl.) F.M.Bailey	RBG Tasmania, Hobart	-12.94 ± 0.70
Cupressaceae	*Callitris glaucophylla* [a]	Joy Thomps. & L.A.S.Johnson	RBG Mount Annan, 873453	-15.30 ± 0.36
Cupressaceae	*Callitris gracilis* [ac]	R.T.Baker	University of Tasmania, Hobart	-12.26 ± 0.59
Cupressaceae	*Callitris intratropica* [a]	R.T.Baker & H.G.Smith	RBG Sydney	-12.81 ± 0.73
Cupressaceae	*Callitris oblonga* [a]	Rich. & A.Rich.	Bedgeburry National Pinetum, 22-0229	-10.88 ± 0.85
Cupressaceae	*Callitris preissii* [ac]	Miq.	University of Tasmania, Hobart	-14.97 ± 0.50
Cupressaceae	*Callitris rhomboidea* [abc]	R.Br. ex Rich & A.Rich.	University of Tasmania, Hobart	-10.32 ± 0.53
Cupressaceae	*Calocedrus formosana* [a]	(Florin) Florin	Bedgeburry National Pinetum, 22-279	-4.92 ± 0.65
Cupressaceae	*Chamaecyparis obtusa* [ac]	(Siebold & Zucc.) Endl.	RBG Kew, 1969-10594	-3.71 ± 0.12
Cupressaceae	*Chamaecyparis pisifera* [ac]	(Siebold & Zucc.) Endl.	RBG Kew, 607-12-60702	-3.46 ± 0.21
Cupressaceae	*Cryptomeria japonica* [a]	(Thunb. ex Lf.) D.Don	Japan	-3.66 ± 0.16
Cupressaceae	*Cunninghamia lanceolata* [a]	(Lamb.) Hook.	RBG Kew, 1973-16525	-3.50 ± 0.17
Cupressaceae	*x Cupressocyparis leylandii* [a]	(A.B.Jacks. & Dallim.) Dallim.	RBG Kew	-8.58 ± 0.17
Cupressaceae	*Cupressus dupreziana* [ac]	A.Camus	RBG Kew, 1970-61193	-10.29 ± 0.06
Cupressaceae	*Cupressus funebris* [a]	Endl.	Bedgeburry National Pinetum, 21-0595	-10.63 ± 0.61
Cupressaceae	*Cupressus glabra* [ab]	Sudw.	France	-11.32 ± 1.03
Cupressaceae	*Cupressus macrocarpa* [a]	Hartw.	Bedgeburry National Pinetum, 29-0407	-6.73 ± 0.38
Cupressaceae	*Cupressus sempervirens* [ab]	L.	France	-10.39 ± 1.10
Cupressaceae	*Cupressus torulosa* [ac]	D.Don	RBG Kew, 1996-1799	-8.35 ± 0.59
Cupressaceae	*Diselma archeri* [a]	Hook.f.	RBG Tasmania, Hobart	-8.72 ± 0.54
Cupressaceae	*Fitzroya cupressoides* [a]	(Molina) I.M.Johnst.	Argentina	-5.00 ± 0.37

Cupressaceae	*Juniperus chinensis* [a]	L.	Bedgeburry National Pinetum , 03-4	-10.88 ± 0.54
Cupressaceae	*Juniperus communis* [ab]	L.	France	-6.37 ± 0.22
Cupressaceae	*Juniperus osteosperma* [ab]	(Torr.) Little	Utah, USA	-8.68 ± 0.35
Cupressaceae	*Juniperus scopulorum* [ab]	Sarg.	Montana, USA	-9.83 ± 0.30
Cupressaceae	*Metasequoia glyptostroboides* [bc]	Hu & W.C.Cheng	RBG Kew, 1980-6256	-2.91 ± 0.13
Cupressaceae	*Papuacedrus papuana* [a]	(F.Muell.) H.L.Li	RBG Sydney, 20114	-4.69 ± 0.23
Cupressaceae	*Platycladus orientalis* [ac]	(L.) Franco	RBG Kew, 1976-3574	-9.04 ± 0.45
Cupressaceae	*Sequoia sempervirens* [abc]	(D.Don) Endl.	University of Bordeaux, Château du Haut-carré	-4.38 ± 0.17
Cupressaceae	*Sequoiadendron giganteum* [abc]	(Lindl.) J.Buchholz	University of Bordeaux, Château du Haut-carré	-3.78 ± 0.06
Cupressaceae	*Taiwania cryptomerioides* [ac]	Hayata	RBG Kew, 1994-900	-3.38 ± 0.29
Cupressaceae	*Taxodium distichum* [ab]	(L.) Rich.	France	-2.29 ± 0.07
Cupressaceae	*Taxodium mucronatum* [a]	Ten.	RBG Sydney, 2005973	-2.23 ± 0.11
Cupressaceae	*Tetraclinis articulata* [a]	(Vahl) Mast.	RBG Sydney, 940902	-13.21 ± 0.75
Cupressaceae	*Thuja plicata* [abc]	Donn ex D.Don	RBG Kew, 1973-18600	-4.20 ± 0.13
Cupressaceae	*Thujopsis dolabrata* [ac]	(L.f.) Siebold & Zucc.	RBG Kew, 1969-16072	-4.15 ± 0.38
Cupressaceae	*Widdringtonia nodiflora* [a]	(L.) E.Powrie	RBG Tasmania, Hobart	-7.87 ± 0.49
Cupressaceae	*Xanthocyparis nootkatensis* [ac]	(D.Don) Farjon & D.K.Harder	RBG Kew, 1969-13806	-5.13 ±0.25
Pinaceae	*Abies alba* [ab]	Mill.	France	-4.00 ± 0.11
Pinaceae	*Abies balsamea* [c]	(L.) Mill.	University of Alberta, Edmonton, Canada	-3.64 ± 0.34
Pinaceae	*Abies forrestii* [a]	Craib	RBG Kew, 1993-1445	-3.42 ± 0.07
Pinaceae	*Abies grandis* [ab]	(Douglas ex D.Don) Lindl.	Idaho, USA	-3.65 ± 0.06
Pinaceae	*Abies lasiocarpa* [ab]	(Hook.) Nutt.	Idaho, USA	-3.62 ± 0.07
Pinaceae	*Abies pinsapo* [ab]	Boiss.	France	-4.15 ± 0.14
Pinaceae	*Abies sachalinensis* [a]	Mast.	Hokkaido, Japan	-3.23 ± 0.07
Pinaceae	*Cedrus atlantica* [abc]	(Endl.) G.Manetti ex Carrière	RBG Kew, 2000-4686	-5.13 ± 0.08
Pinaceae	*Cedrus deodara* [bc]	(Roxb. ex D.Don) G.Don	Clermont-Ferrand, France	-6.69 ± 0.36
Pinaceae	*Larix decidua* [abc]	Mill.	RBG Kew, 1979-6300	-4.11 ± 0.27
Pinaceae	*Larix gmelinii* [a]	(Rupr.) Kuzen.	Bedgeburry National Pinetum, 07-0095	-3.13 ± 0.18
Pinaceae	*Larix occidentalis* [ab]	Nutt.	Idaho, USA	-4.21 ± 0.14
Pinaceae	*Picea abies* [b]	(L.) H.Karst.	France	-3.66 ± 0.09
Pinaceae	*Picea engelmannii* [b]	Parry ex Engelm.	Montana, USA	-4.18 ± 0.09
Pinaceae	*Picea glauca* [c]	(Moench) Voss	University of Alberta, Edmonton, Canada	-4.35 ± 0.26
Pinaceae	*Picea likiangensis* [a]	(Franch.) E.Pritz.	RBG Kew, 1970-6137	-3.86 ± 0.13
Pinaceae	*Picea mariana* [c]	(Mill.) BSP.	Edson, Canada	-5.21 ± 0.19
Pinaceae	*Pinus albicaulis* [ab]	Engelm.	Montana, USA	-3.19 ± 0.10
Pinaceae	*Pinus cembra* [ab]	L.	Austria, Europe	-3.02 ± 0.17
Pinaceae	*Pinus contorta* [b]	Douglas ex Loudon	Montana, USA	-3.90 ± 0.18
Pinaceae	*Pinus edulis* [b]	Engelm.	Utah, USA	-4.03 ± 0.06
Pinaceae	*Pinus flexilis* [ab]	E.James	Montana, USA	-3.71 ± 0.18
Pinaceae	*Pinus halepensis* [ab]	Mill.	France	-4.67 ± 0.05
Pinaceae	*Pinus hartwegii* [c]	Lindl.	RBG Kew, 1996-1016	-3.42 ± 0.05
Pinaceae	*Pinus mugo* [ab]	Turra	Austria, Europe	-3.74 ± 0.07
Pinaceae	*Pinus nigra* [c]	J.F.Arnold	RBG Kew, 1973-15503	-3.52
Pinaceae	*Pinus pinaster* [abc]	Aiton	Bordeaux, France, 503, 361, 441, 463B	-3.72 ± 0.07

Pinaceae	*Pinus pinea* [ab]	L.	France	-4.34 ± 0.16
Pinaceae	*Pinus ponderosa* [ab]	Douglas ex Loudon	Montana, USA	-3.86 ± 0.05
Pinaceae	*Pinus radiata* [c]	D.Don	University of Tasmania, Hobart	-4.37 ± 0.14
Pinaceae	*Pinus sylvestris* [b]	Frankis ex Businsky	France	-3.20 ± 0.02
Pinaceae	*Pinus uncinata* [ab]	Ramond ex DC.	France	-0.17 ± 0.17
Pinaceae	*Pinus wallichiana* [c]	A.B.Jacks.	RBG Kew, 1979-2373	-2.83 ± 0.11
Pinaceae	*Pseudolarix amabilis* [a]	(J.Nelson) Rehder	RBG Kew, 1960-13101	-4.16 ± 0.15
Pinaceae	*Pseudotsuga menziesii* [ab]	(Mirb.) Franco	Montana, USA	-3.68 ± 0.15
Podocarpus	*Acmopyle pancheri* [a]	(Brongn. & Gris) Pilg.	RBG Tasmania, Hobart	-3.62 ± 0.07
Podocarpus	*Afrocarpus gracilior* [a]	(Pilg.) C.N.Page	RBG Tasmania, Hobart, 10128	-6.36 ± 0.10
Podocarpus	*Dacrycarpus dacrydioides* [b]	(A.Rich.) de Laub.	Australia	-2.51 ± 0.16
Podocarpus	*Dacrydium araucarioides* [a]	Brongn. & Gris	Noumea, New Caledonia	-3.78 ± 0.39
Podocarpus	*Falcatifolium taxoides* [a]	(Brongn. & Gris) de Laub.	RBG Tasmania, Hobart	-5.55 ± 0.53
Podocarpus	*Halocarpus bidwillii* [a]	(Hook.f. ex Kirk) Quinn	Bedgeburry National Pinetum, 14-465	-5.35 ± 0.21
Podocarpus	*Lagarostrobos franklinii* [a]	(Hook.f.) Quinn	RBG Kew, 14-53	-4.35 ± 0.36
Podocarpus	*Manoao colensoi* [a]	(Hook.) Molloy	RBG Tasmania, Hobart, 40838	-2.88 ± 0.21
Podocarpus	*Phyllocladus trichomanoides* [a]	D.Don	Bedgeburry National Pinetum, 17-0386	-7.10 ± 0.25
Podocarpus	*Podocarpus elatus* [a]	R.Br ex Endl.	RBG Sydney, 20040141	-6.74 ± 0.39
Podocarpus	*Podocarpus lawrencei* [a]	Hook.f.	Bedgeburry National Pinetum, 14-464	-3.82 ± 0.21
Podocarpus	*Podocarpus rubens* [a]	de Laub.	RBG Tasmania, Hobart, 10116	-3.73 ± 0.04
Podocarpus	*Podocarpus salignus* [a]	D.Don	Bedgeburry National Pinetum, 1993-1660	-4.28 ± 0.16
Podocarpus	*Podocarpus spinulosus* [a]	(Sm.) R.Br ex Mirb.	RBG Sydney, 990828	-6.84 ± 0.34
Podocarpus	*Podocarpus totara* [a]	G.Benn. ex D.Don	Wakehurst, 2009-2494	-4.95 ± 0.16
Podocarpus	*Prumnopitys ladei* [a]	(F.M.Bailey) de Laub.	RBG Sydney, 822906	-6.68 ± 0.23
Podocarpus	*Retrophyllum comptonii* [a]	(J.Buchholz) C.N.Page	RBG Tasmania, Hobart, 99069	-2.54 ± 0.09
Podocarpus	*Saxegothaea conspicua* [a]	Lindl.	Argentina	-3.39 ± 0.23
Podocarpus	*Sundacarpus amarus* [a]	(Blume) C.N.Page	RBG Tasmania, Hobart, 1331	-2.83 ± 0.14
Sciadopityaceae	*Sciadopitys verticillata* [ac]	Siebold & Zucc.	RBG Kew, 1979-48	-4.07 ± 0.10
Taxaceae	*Taxus baccata* [ab]	L.	France	-6.49 ± 0.31
Taxaceae	*Taxus brevifolia* [ab]	Nutt.	Montana, USA	-6.44 ± 0.30
Taxaceae	*Torreya californica* [ac]	Torr.	RBG Kew, 1969-14196	-6.39 ± 0.30
Taxaceae	*Torreya grandis (fortune)* [ac]	Fortune ex. Lindl.	RBG Kew, 1973-20815	-4.69 ± 0.25
Taxaceae	*Torreya nucifera* [ac]	(L.) Siebold & Zucc.	RBG Kew, 1969-15523	-5.95 ± 0.30

Table S2.

	**Pearson correlations**			
	P_{50}	P_{12}	P_{88}	slope
D_{MP}	0.21	0.25	0.18	0.2
D_{MPmax}	0.05	0.03	0.07	0.06
D_{PM}	0.12	0.18	0.08	0.11
D_{T}	-0.17	-0.17	-0.17	0.01
D_{TO}	0.06	0.16	0.0008	0.36***
N_{MP}	0.22	0.19	0.24	-0.23
P_{WI}	-0.51***	-0.46***	-0.51***	0.38**
T_{TW}	0.15	0.14	0.15	-0.19
(T_{TW}/D_{T})	0.41***	0.36**	0.42***	-0.3**
	**Spearman correlations**			
	P_{50}	P_{12}	P_{88}	slope
D_{PA}	-0.3**	-0.13	-0.39***	0.46***
D_{H}	-0.31**	-0.20	-0.31**	0.26*
F	-0.01	-0.21*	0.12	-0.26*
O	0.46***	0.5***	0.4***	-0.3**
P_{MC}	0.01	0.11	-0.03	0.03
P_{RS}	-0.43***	-0.34**	-0.47***	0.43***
P_{TC}	0.52*	0.55*	0.42	-0.31
P_{TD}	0.12	-0.14	0.3*	-0.41***
R_{MP}	0.09	-0.04	0.15	-0.17
R_{PA}	0.3**	0.1	0.41***	-0.49***
R_{P}	0.47**	0.33	0.58***	-0.54***
V_{EF}	0.52***	0.5***	0.5***	-0.4***

Figure S1. Relationship between A) cavitation resistance (P_{50}) and rupture stretching pressure, B) xylem air entry pressure (P_{12}) and torus deflection pressure and C) between margo capillary-seeding pressure and torus deflection pressure.

Figure S2. Relationship between torus capillary-seeding pressure and cavitation resistance (P_{50}).

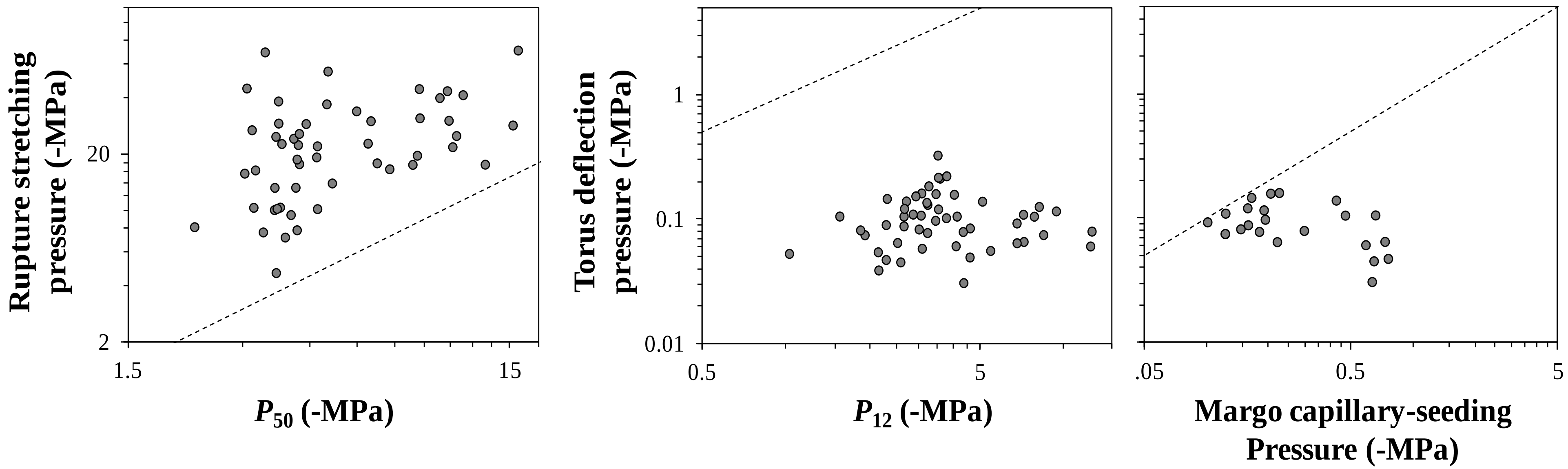

Figure S1. Relationship between A) cavitation resistance (P_{50}) and rupture stretching pressure, B) xylem air entry pressure (P_{12}) and torus deflection pressure and C) between margo capillary-seeding pressure and torus deflection pressure.

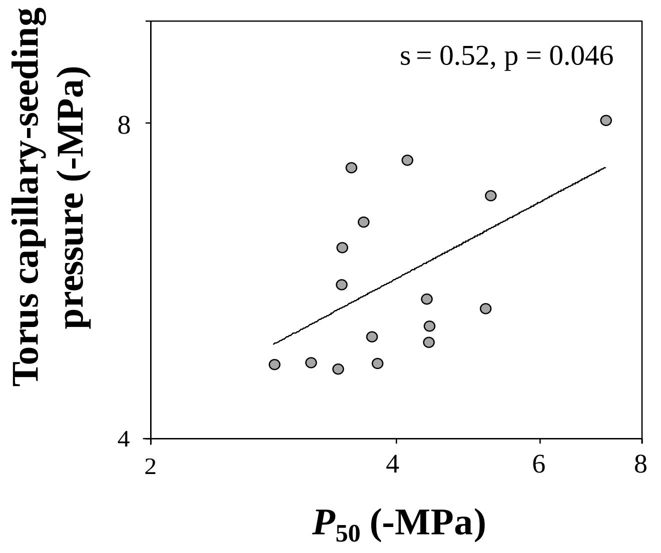

Figure S2. Relationship between torus capillary-seeding pressure and cavitation resistance (P_{50}).
